# Afadin loss induces breast cancer metastasis through destabilisation of E‐cadherin to F‐actin linkage

**DOI:** 10.1002/path.6394

**Published:** 2025-03-03

**Authors:** Max AK Rätze, Lotte NFL Enserink, Noboru Ishiyama, Sven van Kempen, Christina HJ Veltman, Isaac J Nijman, Wisse E Haakma, Carlos Caldas, René Bernards, Paul J van Diest, Matthias Christgen, Thijs Koorman, Patrick WB Derksen

**Affiliations:** ^1^ Department of Pathology University Medical Center Utrecht Utrecht The Netherlands; ^2^ Launchpad Therapeutics, Inc Cambridge MA USA; ^3^ Center for Molecular Medicine, Cancer Genomics Netherlands, Department of Genetics University Medical Center Utrecht Utrecht The Netherlands; ^4^ Cancer Research UK Cambridge Institute University of Cambridge Cambridge UK; ^5^ Department of Oncology University of Cambridge Cambridge UK; ^6^ Division of Molecular Carcinogenesis, Center for Biomedical Genetics and Cancer Genomics Centre The Netherlands Cancer Institute Amsterdam The Netherlands; ^7^ Institute of Pathology Hannover Medical School Hannover Germany

**Keywords:** metastatic breast cancer, Afadin, E‐cadherin, adherens junction

## Abstract

Afadin is a multimodal scaffolding protein with essential functions in cell–cell adhesion. Although its loss of expression has been linked to breast cancer invasion and metastasis, the underlying mechanisms driving tumour progression upon mutational Afadin (*AFDN*) loss in breast cancers remains unclear. In the current study we identified a somatic frameshift *AFDN* mutation (*p*.Lys630fs) in an invasive breast cancer sample that coincides with loss of Afadin protein expression. Functional studies in E‐cadherin‐expressing breast cancer cells show that Afadin loss leads to immature and aberrant adherens junction (AJ) formation. The lack of AJ maturation results in a noncohesive cellular phenotype accompanied by Actomyosin‐dependent anoikis resistance, which are classical progression hallmarks of single‐cell breast cancer invasion. Reconstitution experiments using Afadin truncates show that proper F‐actin organisation and epithelial cell–cell adhesion critically depend on the Coiled‐Coil domain of Afadin but not on the designated C‐terminal F‐actin binding domain. Mouse xenograft experiments based on cell lines and primary patient‐derived breast cancer organoids demonstrate that Afadin loss induces single‐cell lobular‐type invasion phenotypes and overt dissemination to the lungs and the peritoneum. In short, Afadin is a metastasis suppressor for breast cancer through stabilisation and maturation of a mechanical E‐cadherin to F‐actin outside‐in link. © 2025 The Author(s). *The Journal of Pathology* published by John Wiley & Sons Ltd on behalf of The Pathological Society of Great Britain and Ireland.

## Introduction

The epithelial adherens junction (AJ) is essential for cell–cell connections between two neighbouring cells. Extracellular E‐cadherin (CDH1) homotypic interactions *in cis* and *in trans* provide a mechanical signalling hub within the AJ through linkage to the actin cytoskeleton [1]. Upon cell–cell contact, the cytosolic E‐cadherin tail interacts with p120‐catenin (CTNND1), β‐catenin (CTNNB1), and αE‐catenin (CTNNA1, α‐catenin from hereon), proteins that are essential for the stability of the AJ and for mechanical and biochemical signal transduction towards F‐actin respectively [2]. Linking of the cytoskeleton occurs by indirect binding through either classical Cadherins or Nectins via their associated accessory proteins. Connections to the F‐actin cytoskeleton are dependent on binding to β‐catenin and the subsequent interaction with α‐catenin, facilitating mechanically transduced cues via the F‐actin cytoskeleton and its dynamics [3]. Interestingly, interaction of α‐catenin with the F‐actin cytoskeleton is dependent on interactions with additional proteins, such as Vinculin, to build up the initial forces along the F‐actin cytoskeleton, and to modulate the F‐actin cytoskeleton in response to mechanical forces applied to the AJ [4, 5]. In the past decade, loss of α‐catenin has been advocated as an alternative mode for tumour promotion in malignancies that are caused by E‐cadherin dysfunction, such as invasive lobular breast cancer (ILC) and diffuse gastric cancer [6, 7]. Functional follow‐up studies in breast cancer subsequently showed that α‐catenin loss indeed induces acquisition of Actomyosin‐dependent anoikis resistance and the formation of ILC‐like and mixed ductal and lobular carcinoma (mDLC) phenotypes in cultured cells and in mice [8]. Taken together, these findings indicate that loss of components that function in cell–cell contacts, other than E‐cadherin, can cause destabilisation of the AJ and underpin subsequent tumour aetiology and/or progression.

E‐cadherin and Nectin both control linkage to the F‐actin cytoskeleton. Unlike E‐cadherin, Nectin mediates F‐actin modulation primarily through Afadin (*AFDN*; previously named *MLLT4*) (reviewed in: [9]). The longer l‐Afadin splice variant (hereafter Afadin) controls these connections as a multidomain scaffolding protein that interacts with various tight junction (TJ) components, indirect connections through α‐catenin and the AJ [10], or direct interaction with F‐actin via its Coiled Coil (CC) domain and/or F‐actin Binding (FAB) domain [11, 12, 13]. Moreover, binding of Afadin with LMO7 and ADIP allows for interaction of Nectin‐based junctions with Cadherin‐based AJs [14, 15]. Cell–cell interactions are additionally reinforced by the formation of TJs, multiprotein junctional complexes that are essential in the formation of a functional barrier in epithelium and endothelium and correct apical polarisation of cells [16]. Like the AJ, TJs facilitate cell–cell interactions through a complex arrangement of transmembrane proteins that interact cytoplasmic adaptors such as Zona Occludens proteins [17]. Zona Occludens 1 (ZO1) links the TJ to the AJ through complex formation with α‐catenin and recruitment of JAMA to the Nectin‐based junctions [18, 19]. As such, proteins within the TJ may be critical to the tumour suppressive function of the AJ. Indeed, inactivation of the TJ polarity protein PAR3 induces destabilisation of AJs, leading to disrupted actin dynamics and invasion of breast cancer cells [20].

In breast cancer, invasive lobular carcinoma (ILC) represents the archetype of single‐cell invading carcinomas. ILC constitutes up to 15% of all breast cancers and is charactersed by noncohesive growth patterns and diffuse dissemination to specific sites such as the gastrointestinal tract, ovaries, and peritoneum [21]. Although it has been established that ILC development and progression are caused by a functional inactivation of E‐cadherin (*CDH1*) [22, 23, 24], up to 10% of ILC cases retain E‐cadherin expression [25], and roughly 30% lose E‐cadherin protein expression without detectable *CDH1* inactivating mutations [26]. These findings imply that alternative drivers exist that confer E‐cadherin‐dependent metastasis suppression in breast cancer.

Here we performed targeted sequencing on primary breast cancers to reveal alternative drivers of breast cancer invasion and the ILC tumour type. We identified Afadin as a candidate tumour suppressor for the development and progression of breast cancer through detrimental modulation of E‐cadherin and the F‐actin cytoskeleton.

## Materials and methods

### Patient material and adhesome sequencing

DNA extracted from ILC samples was kindly provided by the RATHER consortium [27]. Processed DNA was captured using a custom Agilent SurePrint array as previously described, based on the Adhesome as listed in supplementary material, Table S1. Sequencing of enriched samples was performed using an Illumina Miseq (San Diego, CA, USA) sequencer to a depth of >50× average target base coverage. All sequencing data were analysed for mutations using in‐house‐developed and validated pipelines [28, 29, 30, 31]. All mutations were mapped to GRCh37.

### Immunofluorescence

MCF7 cells were allowed to adhere to glass coverslips overnight, washed with phosphate‐buffered saline (PBS) containing Ca^2+^ and Mg^2+^ (Catalogue number 14080055, Gibco, Paisley, Scotland, UK) and subsequently fixed using 4% paraformaldehyde. Samples were blocked using 4% bovine serum albumin (BSA) in PBS for 1 h. For the 209 T organoid line (HUB‐01‐C2‐156 [32]) organoids were allowed to form in BME (3432‐001‐01; R&D Systems, Minneapolis, MN, USA), after which the organoids were fixed using 4% paraformaldehyde supplemented with 0.1% glutaraldehyde (49629; Merck, Darmstadt, Germany) for 15 min. After washing, samples were then incubated with 1% NaBH_4_ (Sigma, St. Louis, MO, USA) for 2 × 15 min. Finally, samples were blocked for 16 h using 5% Normal Goat Serum (ThermoFisher, Waltham, MA, USA) and 0.3% Triton X‐100. Both cell‐line and organoid samples were incubated with primary antibodies overnight at 4 °C. The following primary antibodies were used: mouse anti‐p120 6H11 (1:500; Santa Cruz Biotechnology, Dallas, TX, USA), rabbit anti‐GFP (1:1000; FL Santa Cruz Biotechnology), rabbit anti‐l‐Afadin (1:500, Sigma‐Aldrich, Zwijndrecht, The Netherlands), monoclonal rat anti‐E‐cadherin (DECMA‐1, 1:300, Sigma‐Aldrich), mouse anti‐p120‐catenin (1:400, BD Biosciences, Franklin Lakes, NJ, USA, clone98/pp120), mouse anti‐α‐catenin (1:100, Enzo Life Sciences, Farmingdale, NY, USA). For detection, samples were incubated with secondary antibodies in blocking buffer for 1 h at room temperature. The following secondary antibodies from Invitrogen (La Jolla, CA, USA) were used at 1:600: goat anti‐mouse Alexa^488^ (#A11029), goat anti‐rabbit Alexa^488^ (#A11034), goat anti‐mouse Alexa^568^ (#A11031), goat anti‐rabbit Alexa^568^ (#A11036), goat anti‐rabbit Alexa^647^ (#A21245), and goat anti‐rat Alexa^647^ (#A21247). Afterwards, cells were incubated with Alexa^633^‐phalloidin to visualise F‐actin (1:300; #A22284; Life Technologies, Carlsbad, CA, USA) or DAPI to stain DNA, washed, and mounted using Prolong Diamond Antifade Mountant (P36961; ThermoFisher Scientific). Imaging was performed using either a LSM700 confocal microscope (MCF7) or a LSM880 for the organoid 209T line (Zeiss, Jena, Germany). Image processing was performed on at least six independent experiments and processed/quantified using ImageJ (v. 1.53b; National Institutes of Health, Bethesda, MD, USA), Illustrator, and Photoshop (v. 2024; Adobe, San José, CA, USA).

### Histological analysis and immunohistochemistry

Formaldehyde‐fixed, paraffin‐embedded tissues were sectioned at 4‐μm and stained with haematoxylin and eosin (H&E). For immunohistochemical staining, fixed sections were rehydrated and incubated with rabbit primary antibodies against ER (SP1; 1:1; Roche, Basel, Switzerland), GATA3 (L50‐823; 1:1; Roche), E‐cadherin (ECH6, 1:600, Zytomed, Langenzersdorf, Germany), and GFP (SC‐8334;1:150; Santa Cruz Biotechnology). Endogenous peroxidases were blocked with 3%v/v H_2_O_2_, followed by biotin‐conjugated secondary antibodies and by incubation with HRP‐conjugated streptavidin–biotin complex (Dako, Carpinteria, CA, USA). Colour was developed using DAB (Dako) and pictures were produced using a Nikon Eclipse E800microscope with a Nikon DXM1200 digital camera (Nikon, Amsterdam, The Netherlands). Appropriate positive (expressing tissues) and negative controls (matched isotype and omitting primary antibody) were used throughout.

### Cell culture

MCF7 cells were cultured as described previously [33]. The breast cancer PDO model 209T (HUB‐01‐C2‐156 [32]) was cultured in 3D BME matrix (R&D Systems, cat. no. 3533‐005‐02) as described [34]. Cell models were profiled using short‐tandem repeat (STR) profiling (LGC Standards).

### CRISPR/Cas9‐generated *AFDN* KO baculovirus

Baculoviruses expressing Cas9 were produced as described previously [35]. Two gRNAs were tested and after TIDE analysis the highest efficiency gRNA was expanded [36]. gRNAs and primers used to perform TIDE analysis are listed in supplementary materials, Table S3.

### Anoikis assay

Anoikis resistance was quantified as described previously [37]. Samples were treated with: 10 μm Y‐27632 (Selleckchem, Huissen, The Netherlands),0.02 μg/ml C3‐transferase inhibitor (CT04‐A cytoskeleton Inc, Heerhugowaard, The Netherlands) or 3 μm blebbistatin (203,390; VWR, Radnor, PA, USA), co‐cultured with cells over 4 days in anchorage‐independent conditions using ultra‐low cluster 24 well plates (Corning, Corning, NY, USA). Error bars depict SD. A two‐tailed Students' *t*‐test was performed to calculate statistical significance; *p* values less than 0.05 were considered significant.

### DNA constructs

pEGFP‐Afadin containing the full rat Afadin cDNA was a kind gift from Yoshimi Takai [38]. N‐terminally GFP conjugated Afadin truncates were cloned into a pLV lentiviral backbone (pLV.bc.PURO, [39]) by In‐Fusion PCR cloning (638,917; Takarabio, San José, CA, USA) using the pEGFP‐l‐Afadin plasmid as template.

### Transfections

Cells were plated at a density of 10^6^ per 10‐cm plate and transfected on the following day using Fugene HD (Promega, Madison, WI, USA, E2311). A PGK‐GFP vector was transfected in parallel as the control for transfection efficiency. Three days after transfection, cells were selected for 2 days using 2 μg/ml puromycin (Sigma‐Aldrich, P8833).

### Intraductal injections and mouse studies

MCF7 and 209T PDO cells (250,000) were suspended in 25 μl and injected intraductally in the fourth mammary gland of recipient Rag2^KO^;IL2cγR^KO^ mice (Envigo, Indianapolis, IN, USA). Tumour onset and growth was monitored as described [40]. Animals that developed tumours were euthanized if the tumours reached a size of >1,000 mm^3^ or in cases of severe discomfort otherwise. All animal experiments were performed in accordance with local, National, and European guidelines under permit AVD1150020209964 issued by The Netherlands Food and Consumer Product Safety Authority (NVWA) of the Ministry of Agriculture, Nature and Food. To determine the average cumulative metastatic burden in the lungs, the number of cells were quantified per identified lung metastasis, after which the number of cells per metastasis was averaged. Cell quantification was performed using the Open Software for Bioimage Analysis QuPath (v0.5.1; https://qupath.github.io).

### Immunoblotting

Cells were lysed, western blotted, probed with peroxidase‐conjugated antibodies, and analysed as described [37]. The following primary antibodies were used: Rabbit anti‐l‐Afadin (1:1000, A0349; Sigma‐Aldrich), rabbit anti‐GFP (1:500; sc‐8334, Santa Cruz Biotechnology), and goat anti‐AKT (1:1,000, C‐20/sc‐1618; Santa Cruz Biotechnology).

### cBioportal analysis and data extraction

Data for the METABRIC and TCGA datasets were retrieved from the cBioportal public databases (https://www.cbioportal.org/) [41, 42, 43]. Data for mutual exclusivity were retrieved and analysed from the mutual exclusivity section of cBioportal, querying for both *CDH1* and *AFDN*. All other reported data were exported from the mutations section, querying for *AFDN*.

### Statistical analysis

All statistical tests were performed using Prism 9 v. 9.4.1 (GraphPad, Boston, MA, USA).

## Results

### Identification of alternative driver mutations in single cell invading breast cancer

**Figure 1:**
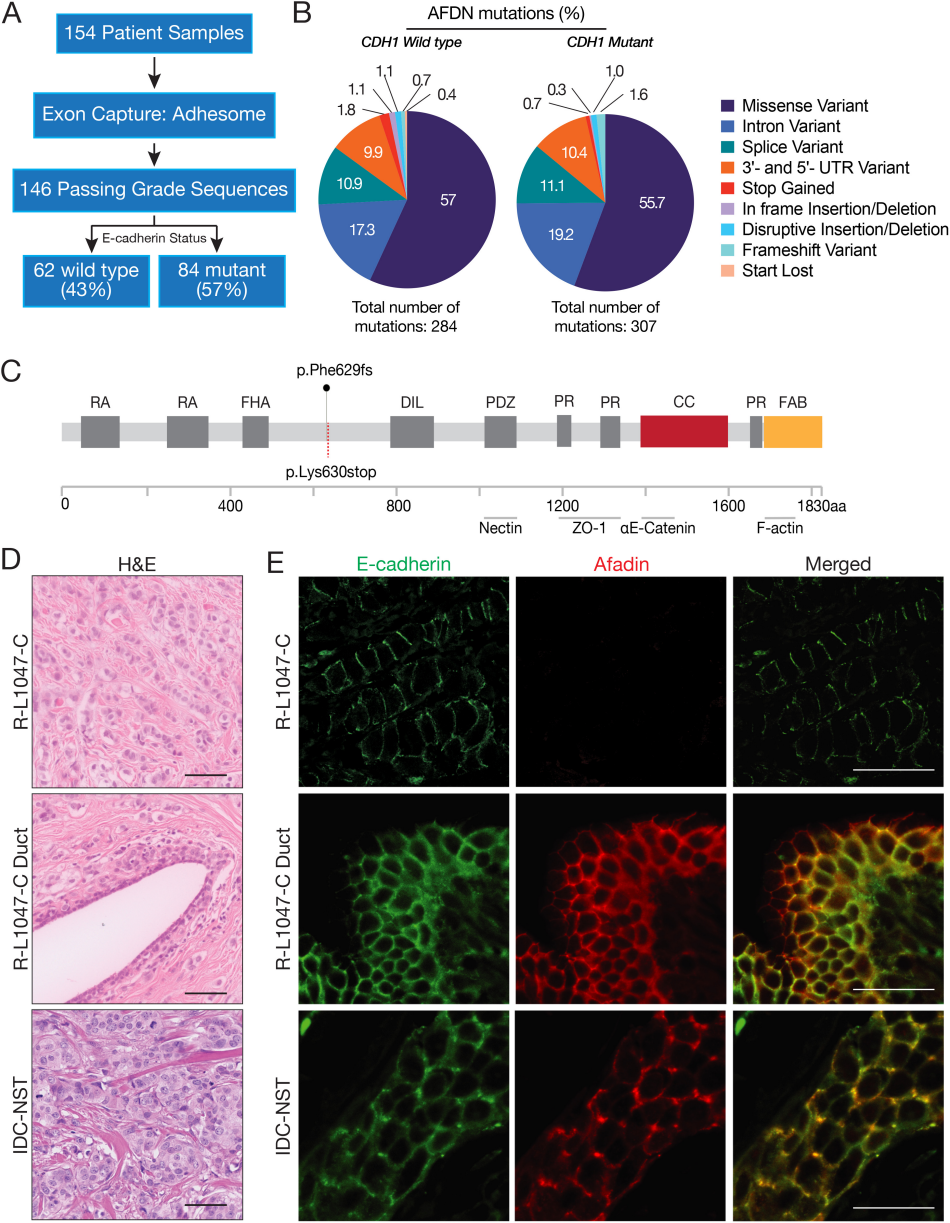
Identification of Adhesome mutations in *CDH1* wildtype patients. (A) Dataflow for the Adhesome analysis in ILC. Indicated are the percentages *CDH1* wildtype versus mutant ILC cases. (B) Distribution of Adhesome mutation types. Pie charts depicting the mutation types found in *CDH1* wildtype and mutant ILC. Numbers represent % of total. (C) Schematic depiction of the Afadin protein. Indicated are the functional domains, the identified frameshift mutation, and the predicted truncating stop codon in patient R‐L1047‐C. Domains and regions: RA, Ras associated; FHA, forkhead associated; DIL, Dilution; PR, proline rich; CC, coiled coil; FAB, F‐actin binding. The binding sites for the effector proteins are indicated. Partly adapted from [44]. (D) Histology from sample R‐L1047‐C (top), a healthy mammary duct with ILC infiltrate from sample R‐L1047‐C (middle), accompanied by a representative invasive ductal carcinoma‐nonspecific type (IDC‐NST) sample (bottom). Scale bar, 50 μm. (E) Afadin protein expression is lost in ILC sample R‐L1047‐C containing the identified *p*.Phe629fs mutation. Shown are immunofluorescence images for E‐cadherin (left) and Afadin (middle) expression. Shown are ILC sample R‐L1047‐C (top), a healthy mammary duct with ILC infiltrate from sample R‐L1047‐C (middle), accompanied by a representative IDC‐NST sample (bottom). Right panels show the merged composite. Scale bar, 50 μm.

To identify alternative drivers of breast cancer progression we performed exome sequencing analysis on 86 genes that play an essential role in epithelial cell–cell adhesion. This gene set (from hereon: the Adhesome) contains genes coding for AJ, TJ, and desmosome (DS) proteins (supplementary material, Table S1). To enable further stratification of genes that control single‐cell invasive breast cancer, we selected 154 ER‐positive, invasive lobular breast cancer (ILC) samples and sequenced the Adhesome genes. Of the 146 samples that passed the sequencing quality checks (Figure 1A), we found mutations causing both deletion of the protein and a disadvantageous outcome. *CDH1* mutations were detected in 84 samples (57.5% of total). To identify novel single‐cell invasion drivers other than E‐cadherin, *CDH1* mutant samples were excluded from further analysis. In the remaining 62 samples, 284 nonsynonymous mutations were identified in a total of 78 different genes (Figure 1B). From this, we prioritised a p.Lys630Stop frameshift mutation in Afadin (*AFDN*), based on its published established roles in cell–cell adhesion [45]. In this breast cancer sample, *AFDN* mutations indeed led to loss of Afadin protein expression (Figure 1C), suggesting classical inactivation of the wildtype allele. The *AFDN* mutant breast cancer sample showed small‐ to medium‐sized breast cancer cells with low mitotic activity, arranged in one to two cell‐thick trabeculae and single‐cell files with targetoid growth patterns (Figure 1D). Importantly, we confirmed that Afadin protein expression was indeed lost in this sample, which coincided with aberrant punctate expression of E‐cadherin at the plasma membrane (Figure 1E). In short, an inactivating mutation in *AFDN* results in loss of protein expression and an E‐cadherin‐positive ILC with a classical morphology.

### Somatic *AFDN* mutations are associated with *CDH1* wildtype lobular breast cancer

We next analysed the occurrence of *AFDN* mutations in METABRIC and TCGA. Overall, 1/142 tumours (0.7%) showed a reported truncating mutation. In total, 79 unique somatic *AFDN* mutations were reported in 88 mammary carcinomas, of which 70 (79.5%) were IDC‐NST (invasive ductal carcinoma, no special type), 10 (11.4%) ILC, and five (5.6%) mixed carcinoma cases (supplementary material, Table S2). The remaining three cases (3.3%) were reported without specified histologic subtype. Among the 54 missense mutations, 34 (63.0%) are associated with decreased *AFDN* mRNA expression (supplementary material, Table S2). Of note, of the 25 *AFDN* truncating (frameshift and nonsense) mutations, 24 (88.0%) are predicted to cause loss of the C‐terminal domain of *AFDN*, leading to loss of the CC domain, including the α‐catenin binding site and putative F‐actin binding region, and the established FAB domain (supplementary material, Figure S1A), potentially impacting E‐cadherin function.

We found significant overall mutual exclusivity for *CDH1* and *AFDN* mutations (*p* < 0.0001; supplementary material, Figure S1B), especially in the subtypes ILC and mixed ductal/lobular carcinoma (*p* < 0.0001; supplementary material, Figure S1B). Blinded histopathological evaluation of 20 TCGA cases containing *AFDN* mutations prompted us to revise two cases that were initially diagnosed as IDC‐NST as a mixed Ductal and Lobular carcinoma (mDLC) (TCGA‐AO‐A128‐01 and TCGA‐B6‐A0X7‐01, supplementary material, Table S2).

### Inactivation of *AFDN* leads to loss of cell–cell contact and anoikis resistance

**Figure 2:**
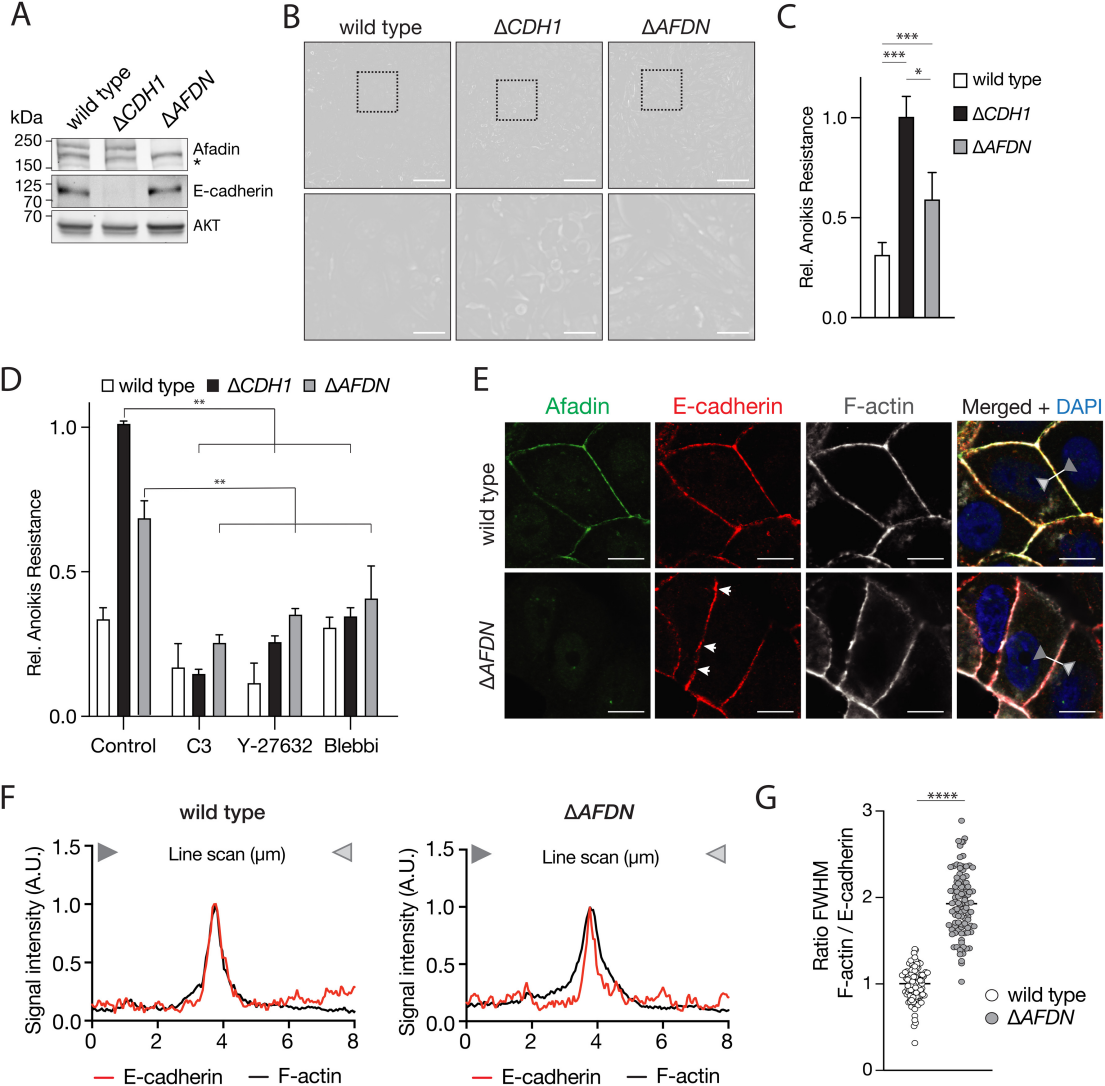
Loss of *AFDN* leads to loss of cell–cell adhesion and anoikis resistance through attenuation of proper AJ formation. (A) Western blot showing the effect of CRISPR‐Cas9 mediated knockout of *AFDN* or *CDH1* in MCF7 cells. AKT was used as a loading control. *, unspecific protein (loading). (B) Representative DIC images showing MCF7 wildtype, MCF7::∆*AFDN*, and MCF7::∆*CDH1* cells, revealing a phenotypical change in cellular morphology upon *CDH1* or *AFDN* knockout. Bottom row shows enlargements of top row. Scale bar, 100 μm top row, Scale bar, 20 μm bottom row. (C) Afadin loss induces anoikis resistance. Samples were stained with FITC‐conjugated Annexin‐V and propidium iodide, analysed using FACS, and fluorescence measured. (D) Anoikis resistance in MCF7::∆*AFDN* cells is RhoA, Rock1 and Actomyosin dependent. Cells were cultured in suspension conditions and treated with C3 transferase (C3, RhoA inhibitor), Y‐27632 (Rock inhibitor), or blebbistatin (blebbi, Myosin ATPase activity). Anoikis resistance was assessed as in (C). (E) Immunofluorescence for Afadin (green), E‐cadherin (red), and F‐actin (white), showing the attenuation of proper AJ formation upon Afadin loss. Arrowheads indicate the occasional aberrant E‐cadherin localization/clustering on Afadin. The right image is a composite of the left panels including a DNA visualisation (merge + DAPI). Scale bar, 5 μm. (F and G) Afadin is required for proper F‐actin organisation at the epithelial AJ. Line scans are shown in (F) comparing the localization of E‐cadherin (red lines) and F‐actin (black lines) perpendicular to the plasma membrane (F). Dark and light grey triangles depict the directionality of the measurement/quantification. Quantification of colocalization was done based on the ratio of the full‐width at half‐maximum (FWHM) of F‐actin over E‐cadherin from (F) comparing wildtype MCF7 (white bullets) versus MCF7::∆*AFDN* cells (grey bullets). (G) *p < 0.05; **p < 0.01 ***p < 0.001. ****p < 0.0001.

We next deleted *AFDN* in E‐cadherin expressing MCF7 cells (Figure 2A). In contrast to wildtype MCF7 cells, MCF7::∆*AFDN* cells grew as noncohesive motile cells (Figure 2B). Furthermore, MCF7::∆*AFDN* cells cultured in suspended conditions acquired anoikis resistance, comparable to E‐cadherin loss in MCF7::*∆CDH1* (Figure 2C). Using the inhibitors C3 and Y27632 to target the proximal Actomyosin activators RhoA or Rock1 and the Myosin ATPase activity inhibitor blebbistatin, we show that the anoikis resistance of MCF7::∆*AFDN* cells critically depends on Rho/Rock‐dependent Actomyosin contraction, comparable to the MCF7::*∆CDH1* cells (Figure 2D). Overall, we conclude that Afadin loss induces an Actomyosin‐dependent anoikis‐resistant phenotype.

### Afadin loss attenuates proper adherens junction formation and F‐actin linkage

In contrast to MCF7 wildtype cells, MCF7::∆*AFDN* cells showed aberrant expression of E‐cadherin on the plasma membrane, similar to the E‐cadherin expression patterns in the *AFDN* mutant ILC sample. Immunofluorescence (IF) for Afadin confirmed the successful knockout (Figure 2E). Concomitant IF for E‐cadherin, β‐catenin, and α‐catenin showed that an AJ was formed on the plasma membrane in MCF7::∆*AFDN* cells, but with a patchy, punctate expression pattern (Figure 2E and supplementary material, Figure S2A,B). In agreement with the published literature [10], we found that Zona Occludens 1 (ZO1) expression was reduced at the tri‐cellular junctions compared to wildtype controls (supplementary material, Figure S2C). Importantly, IF expression analysis showed that cortical F‐actin distribution was less confined at the plasma membrane when compared to MCF wildtype cells (Figure 2E). To quantify these differences, we examined the spatial E‐cadherin/F‐actin expression and distribution at the plasma membrane using perpendicular line scans and calculated the ratio of F‐actin over E‐cadherin expression at the full‐width at half‐maximum height (FWHM). These analyses confirmed that loss of *AFDN* in MCF7::∆*AFDN* cells lead to diffuse distribution of cortical F‐actin parallel to the plasma membrane (Figure 2F,G). Our findings therefore demonstrate that Afadin controls proper linkage from E‐cadherin to the F‐actin cytoskeleton. Furthermore, our work suggests that Aafadin loss in breast cancer cells induces improper F‐actin mechanical tension, leading to constitutive deregulation of Actomyosin contraction, which has been causally linked to anoikis resistance in metastatic breast cancer [8, 39].

### The coiled‐coil domain of Afadin is essential for proper AJ formation

**Figure 3:**
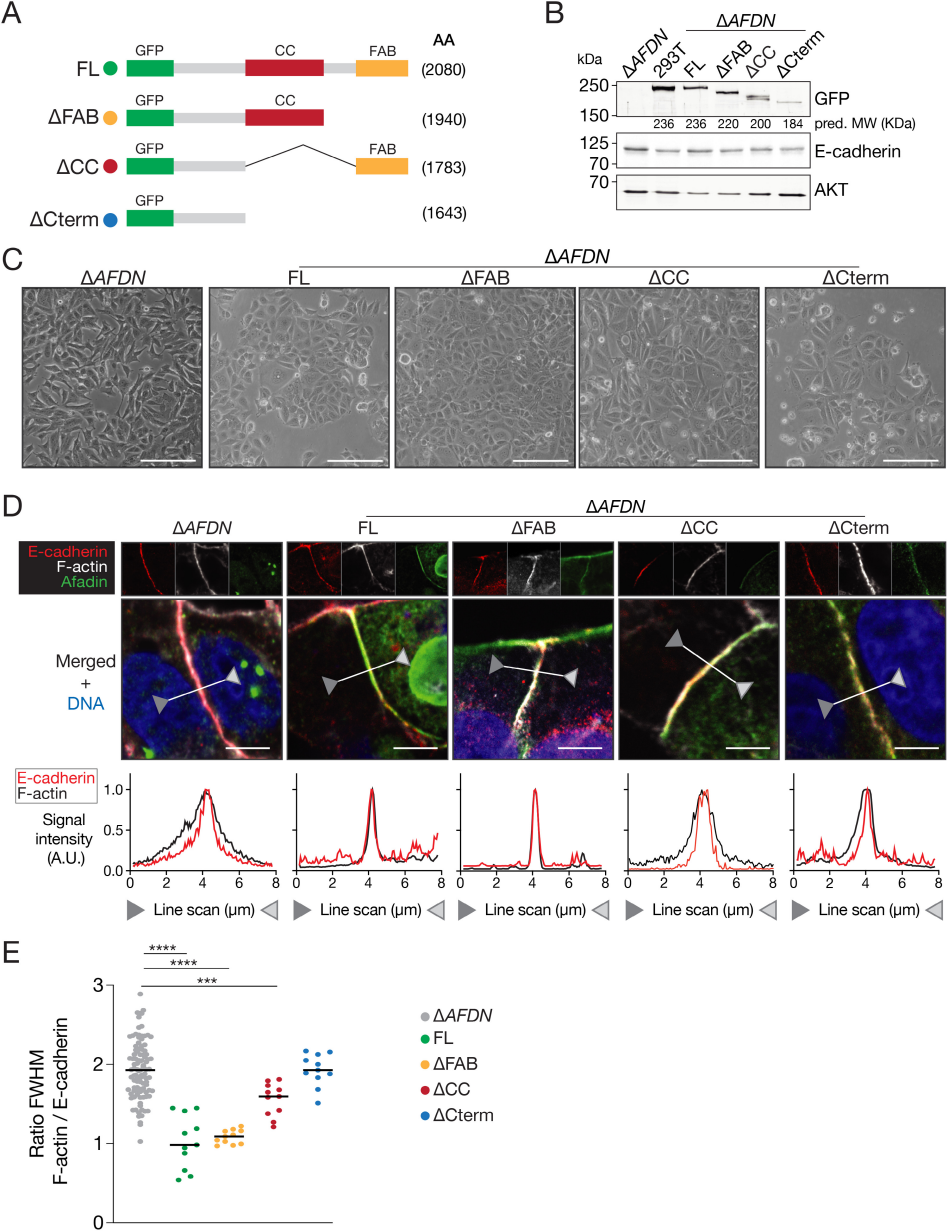
The α‐catenin binding domain of Afadin is essential for epithelial cell–cell adhesion and correct cortical F‐actin localization. (A) Schematic overview of the GFP‐tagged full‐length and truncation Afadin. Number between brackets indicates the number of amino acids (AA). (B and C) Reconstitution of Afadin full‐length or truncate proteins in MCF7::∆*AFDN* cells. (B) Western blotting showing expression based on the GFP tag (top, GFP). The impact of Afadin loss or truncation on E‐cadherin expression was probed (middle), and AKT was used as a loading control (bottom). The predicted molecular protein weight is indicated below the GFP blot (pred. MW). HEK293Ts were used as a transfection control with the FL construct. (C) Representative DIC images showing the phenotypical impact of the afadin truncates in MCF7::∆*AFDN* cells. Similar cell numbers were plated and photographed after 18 h to allow comparisons during optimal expression of the truncates. Scale bars, 10 μm. (D and E) Immunofluorescence images showing MCF7::∆*AFDN* cells reconstituted with the indicated truncates (see Figure 3A for details). E‐cadherin, F‐actin, and GFP‐Afadin were stained (D) and line scans were performed between the arrowheads (indicated in the merged composites). Scale bars, 5 μm. The FWHM ratio of the F‐actin over E‐cadherin expression signals were quantified, normalised to 1, and compared in (E). ***p < 0.001; ****p < 0.0001. Note that the absence of the Afadin F‐actin binding domain is not sufficient to disrupt E‐cadherin to F‐actin linkage, while deletion of the α‐catenin domain is required for proper AJ formation. Images shown are representative examples of at least six independent experiments.

The identified *AFDN* mutations indicate that most truncating mutations lead to loss of the C‐terminal domain, causing impairment of binding to α‐catenin and F‐actin, respectively. We therefore hypothesised that an Afadin‐dependent F‐actin to AJ connection occurs through either binding of α‐catenin to the Afadin CC‐domain, through the Afadin FAB‐domain, or both. To investigate the individual requirement, we used full‐length N‐terminally GFP‐tagged rat l‐Afadin (FL) [38] and generated truncated constructs lacking either the Coiled‐Coil (CC)‐domain containing the α‐catenin binding region (∆CC), the FAB‐domain (∆FAB), or the entire C‐terminus from amino acid position 1,391 onward (∆Cterm) (Figure 3A). We next performed reconstitution experiments in MCF7::∆*AFDN* cells, confirming expression at the correct molecular weight of either the full‐length or truncated constructs using a GFP antibody (Figure 3B). Expression of the FL construct in MCF7::∆*AFDN* cells fully rescued the noncohesive phenotype, resulting in proper cell–cell adhesion and a cobblestone morphology (Figure 3C). In contrast, reconstitution with either the ∆CC or ∆Cterm truncate did not restore the epithelial phenotype in MCF7::∆*AFDN* cells (Figure 3C). Although both truncates did not rescue epithelial morphology, cells reconstituted with ∆CC constructs showed a hypomorphic phenotype, which is a partial retainment of the migratory morphology and spindle‐shaped cells (Figure 3C). Surprisingly, we observed that reconstitution with the ∆FAB construct fully rescues the ∆*AFDN* phenotype (Figure 3C), resulting in proper epithelial cell–cell adhesion comparable to the control MCF7 cells (Figure 2B) and MCF7::FL reconstitution (Figure 3C). In line with the cellular phenotypes, subsequent examination and quantification of the spatial E‐cadherin/F‐actin expression at the plasma membrane confirmed that reconstitution with either the FL or ∆FAB construct fully rescues proper colocalization of cortical F‐actin to E‐cadherin (Figure 3D). In contrast, we observe that neither the ∆CC, nor the ∆Cterm reconstitution can rescue Afadin loss, leading to higher F‐actin/E‐cadherin ratios, that is dispersed cortical F‐actin (Figure 3E).

In short, we show that the coiled‐coil region of Afadin that contains the α‐catenin binding domain is required for proper AJ formation and linkage to the F‐actin cytoskeleton in E‐cadherin‐expressing breast cancer cells. Interestingly, our data indicate that the previously designated ‘F‐actin binding domain’ is indispensable for the formation of proper cell–cell adhesion.

### Loss of  Afadin leads to tumour cell invasion in single files and overt lung metastasis in transplantation‐based mouse models of breast cancer

**Figure 4:**
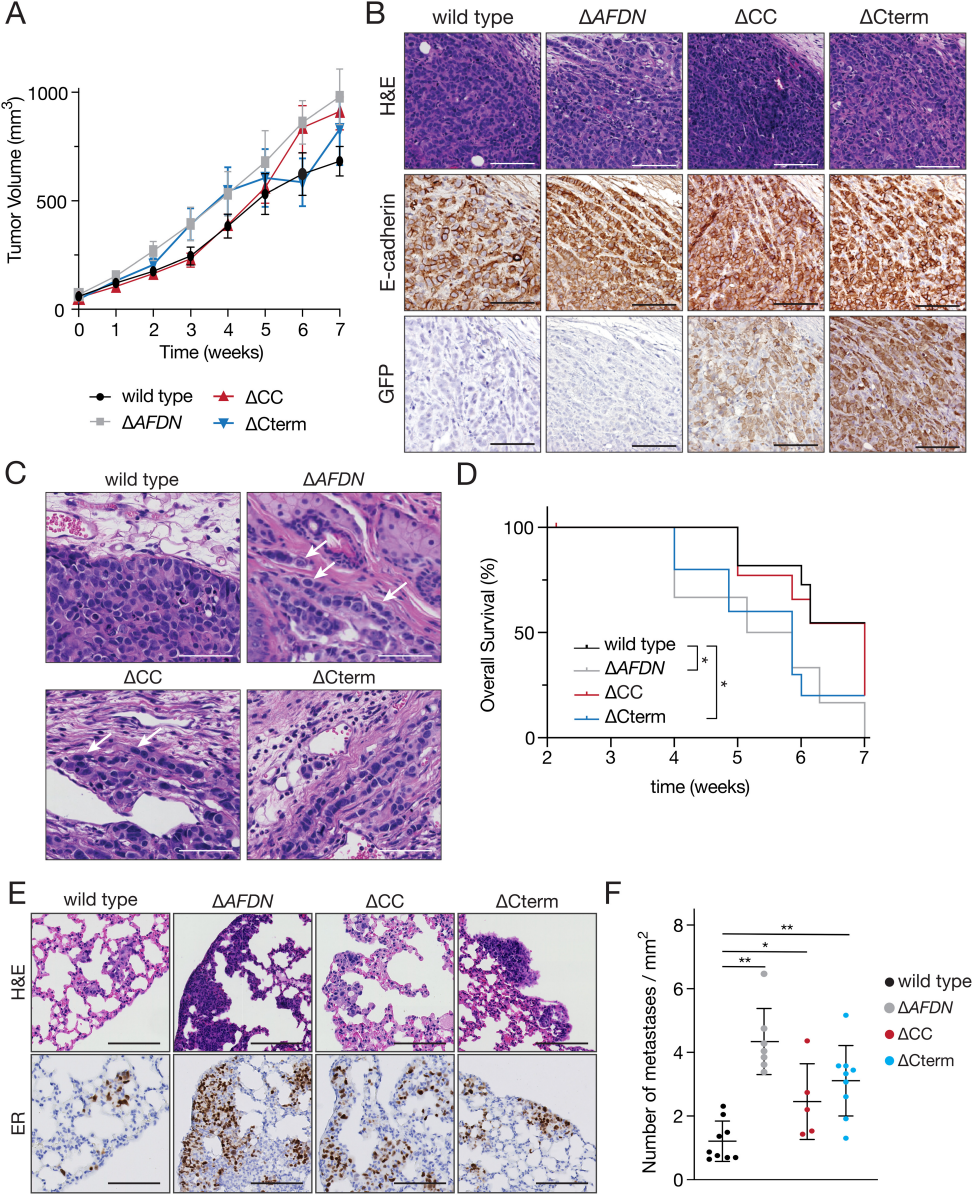
Induction of metastasis requires loss of the α‐catenin binding CC domain of Afadin. (A) Inhibition of proper AJ formation does not impact tumour growth The indicated MCF cells were injected intraductally in mice, and tumour volumes quantified over time. Error bars depict SD. (B) E‐cadherin expression is retained in cells lacking Afadin or Afadin C‐terminal truncates. Shown are immunohistochemistry panels for E‐cadherin (middle) and GFP‐afadin (bottom) in the indicated primary tumours. Left panels are H&E stains. Scale bars, 100 μm. (C) Afadin loss of function induces lobular‐type morphological features. Shown are representative examples of primary tumours that developed in the mice from (A). Note the acquisition of invasive trabecular single cell files (arrows) and individual invading cells (arrowheads). Scale bars, 100 μm. (D) Loss of the Afadin C‐terminal α‐catenin, and F‐actin binding domain results in reduced survival. Shown is a Kaplan–Meier tumour‐free survival plot for the indicated tumour cohorts. Censored cases are marked by ticks on the plots. (E and F) Loss of the Afadin C‐terminal coiled‐coil domain is sufficient to drive metastatic disease. Shown are H&E stains (top) and immunohistochemistry for human oestrogen receptor (ER) (bottom) on lung sections. (E) Scale bars, 50 μm. (F) Quantification of metastases, normalised to lung area. *p < 0.05, **p < 0.01.

To assess the functional impact of *AFDN* loss in breast cancer, we performed mammary intraductal injections using MCF7::∆*AFDN* cells that had been stably reconstituted with either the ∆CC or ∆Cterm GFP‐tagged Afadin truncate. In this setup, we monitored longitudinal tumour volumes and observed no significant differences in primary tumour size between the tumour cohorts, indicating that Afadin loss does not impact primary tumour growth (Figure 4A). All cohorts had retained membranous expression of E‐cadherin (Figure 4B, middle panels) with carcinomas in the rescue cohorts expressing GFP, confirming proper expression of the constructs after transplantation (Figure 4B, bottom panels). Moreover, carcinomas in the wildtype MCF7 cohort were characterised by expansive, circumscribed growth with occasional collective invasion of cells into the duct (Figure 4C). In contrast, mice injected with MCF7::∆*AFDN*, MCF7::∆CC, or MCF7::∆Cterm reconstituted cells showed invasive lobular‐type features, including overt single‐cell invasion and the formation of trabecular and single files, most notably at the invasive front (Figure 4C, white arrows). Interestingly, we observed a significantly shorter overall survival of mice that were injected with either the MCF7::∆*AFDN* knockout cells or the ∆Cterm truncate reconstituted cells (*p* < 0.05) (Figure 4D). This was accompanied by frequent single‐cell intravasation of cancer cells in the vasculature, which was observed in the MCF7::∆*AFDN*, MCF7::∆CC, and MCF7::∆Cterm tumours (Figure 4E). Decreased survival in these mice compared to controls was indeed due to the development of overt lung metastasis of cells expressing human oestrogen receptor (ER), used as a marker for human luminal breast cancer cells (Figure 4F). Although reconstitution with ∆CC did not result in a significant survival decrease, we detected an increase in metastatic burden after quantification of the ER‐positive cells, similar to the increase observed after reconstitution with the ∆Cterm truncate (Figure 4E, F).

To corroborate our findings in primary breast cancer samples, we used a luminal, ER/PR‐expressing and Her2‐negative patient derived breast cancer organoid (PDO) model 209T (HUB‐01‐C2‐156, [32]) and performed CRISPR/Cas9 based knockout of *AFDN* (supplementary material, Figure S3A). When cultured under 3D conditions, *AFDN* knockout induced irregular PDO phenotypes in two independent 209T::∆*AFDN* PDO knockouts (supplementary material, Figure S3B). Subsequent immunofluorescence studies confirmed that Afadin expression was lost in the 209T::∆*AFDN* cells, and that this induced aberrant localization and distribution of E‐cadherin and F‐actin (supplementary material, Figure S3C).

**Figure 5:**
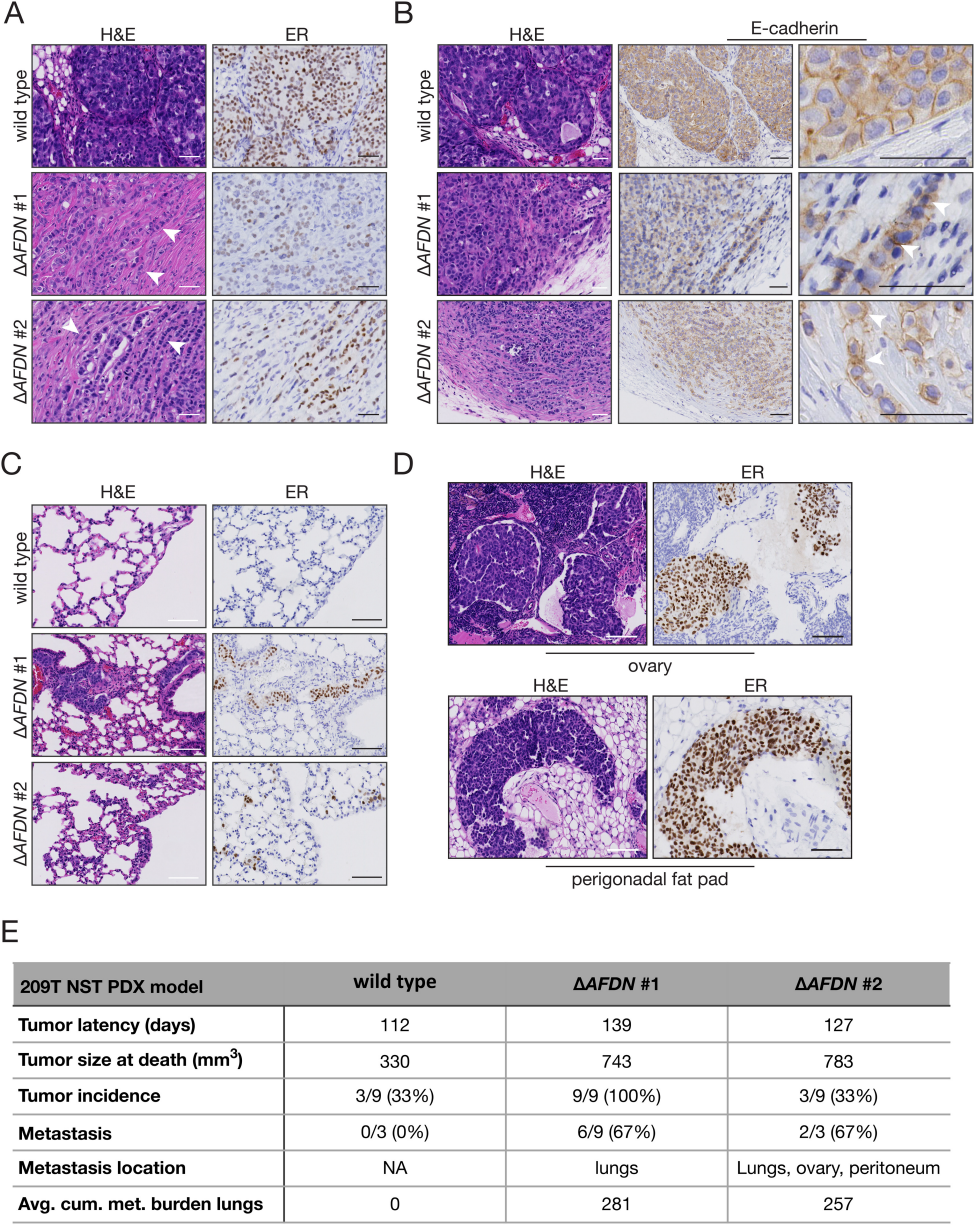
Afadin loss induces metastatic progression in a patient‐derived, ER^POS^/Her^NEG^ organoid xenograft model of luminal breast cancer. (A) Loss of Afadin induces a switch from ductal carcinoma *in situ* (DCIS) to invasive ductal‐lobular (mDLC). *AFDN* knockout was performed in the primary patient‐derived breast cancer organoid (PDO) model 209T (HUB‐01‐C2‐156, [32]) and intraductal mammary gland xenografts using PDO models (PDXO) were done with 209T control (wildtype) and two independent *AFDN* knockout PDO models, 209T::∆*AFDN* #1(∆*AFDN* #1) and 209T::∆*AFDN* #2 (∆*AFDN* #2). Shown are H&E histology (left panels) and ER expression (right panels) displaying the formation of DCIS lesions in the wildtype 209T PDXO (top panels) and invasive mDLC tumours in the 209T PDXO *AFDN* knockout models (∆*AFDN* #1 and ∆*AFDN* #2). Note the formation of single cell invasion (arrowheads). Scale bars, 25 μm. (B) Aberrant E‐cadherin expression upon Afadin loss. Tumours from the indicated models were analysed for E‐cadherin expression using immunohistochemistry. The right panels show enlarged images, depicting aberrant and punctate E‐cadherin expression in the 209T *AFDN* knockout model (arrowheads). Scale bars, 25 μm. (C and D) Afadin induces metastatic disease. Shown are lung sections (C) from the indicated models. Metastatic cells were identified using ER expression. Peritoneal metastatic spread in the PDXO 209T model upon *AFDN* knockout (D). Shown are examples of metastatic dissemination to the ovary and perigonadal fat pad. Scale bars, 50 μm. (E) An overview of tumour growth and metastasis in the 209T NST PDX model. NA = not applicable.

Next, we performed intraductal mammary gland xenografts of control (wildtype 209T) and the two independent Afadin knockout PDO models (209T::∆*AFDN* #1 and 209T::∆*AFDN* #2), which resulted in tumour formation with a median latency of ~120 days. Control 209T PDO xenografted (PDXO) cells produced ductal carcinoma *in situ* (DCIS)‐type lesions in three recipient PDXO animals with an average volume of 330 mm^3^ (Figure 5A,B,E). In contrast, the 209T::∆*AFDN* knockout PDOXs formed larger tumours (average 763 mm^3^), which were diagnosed as mixed ductal‐lobular carcinoma (mDLC), characterised by overt invasion of ER‐expressing cells into surrounding tissues (Figure 5A,B). Moreover, this PDXO model confirmed our previous observations that *AFDN* knockout leads to a punctate and irregular plasma membrane E‐cadherin expression (Figure 5B, bottom panels). Importantly, Afadin loss induced a strong increase in the metastasis spread to the lungs when compared to 209T wildtype tumours (Figure 5C,E). In addition, we observed the formation of peritoneal metastasis in the 209T::∆*AFDN* tumours (Figure 5D), which is a typical trait of diffusely metastasizing breast cancers such as invasive lobular carcinoma.

**Figure 6:**
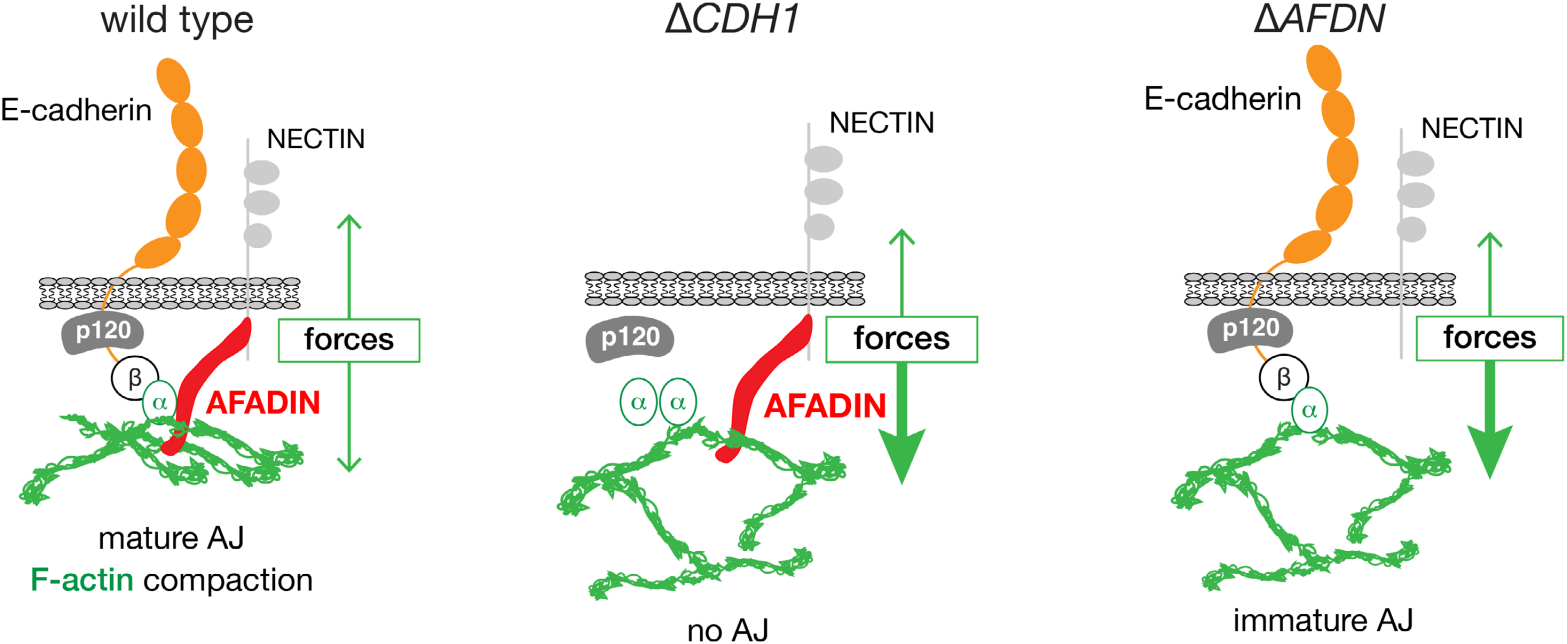
Afadin is essential for invasion suppression of breast cancer. Working model for the consequences of Afadin loss on the interaction of the adherens junction (AJ) and the F‐actin cytoskeleton. In *CDH1* wildtype conditions, the AJ can form proper interactions with the F‐actin cytoskeleton, leading to a balanced outside‐in force distribution from the AJ to the F‐actin cytoskeleton and complete maturation of the AJ. E‐cadherin inactivation causes loss of AJs, leading to a disbalance in mechanical forces from the AJ to the F‐actin cytoskeleton (middle panel). Upon Afadin loss, E‐cadherin‐based AJ are formed, but the lack of stabilisation that occurs from the inability of Afadin to bind the α‐catenin domain containing coiled‐coil region, or failure to establish a dual‐belt F‐actin architecture (i.e. a *zonula adherens matura*), causes a disbalance of forces towards the F‐actin cytoskeleton that prevents proper formation of a mature AJ (right panel). Consequently, cells acquire anoikis resistance driven by Actomyosin contraction and the ability to invade and metastasize.

In summary, we conclude that loss of Afadin expression induces metastatic growth of breast cancer. Afadin inactivation leads to a force imbalance that causes disruption of F‐actin linkage in E‐cadherin‐expressing carcinoma cells (summarised in Figure 6), leading to loss of cell–cell contacts, the acquisition of anoikis resistance, and the progression towards metastatic tumours with lobular features.

## Discussion

Previous data have connected loss of α‐catenin to E‐cadherin expressing lobular‐type cell lines [46] and provided functional evidence that this event leads to mixed ductal/lobular carcinoma (mDLC) lesions that are characterised by and dependent on Actomyosin‐dependent anoikis resistance [8]. Based on these studies, it was proposed that an imbalanced Actomyosin‐dependent force distribution due to loss of proper linkage of E‐cadherin to the F‐actin cytoskeleton may be sufficient to drive single‐cell survival and dissemination during breast cancer progression (reviewed in: [47]). In the current study we used a combination of genomics, cell biology, and mouse experiments to identify Afadin as a metastasis suppressor for lobular‐type breast cancer. Afadin was previously linked to the metastasis‐free survival of breast cancer [48, 49, 50] and recent retrospective phylogenetic analyses identified a *CDH1* wildtype ILC case that presented with an *AFDN* deletion [51]. Interestingly, we found that 82% of the identified *AFDN* mutations in the TCGA and METABRIC databases (0.4% of total) occur in IDC‐NST, which suggests that some cases were potentially misdiagnosed and may in fact histologically be ILC or mDLC. Indeed, from the 20 *AFDN* mutant TCGA cases that had available histology data, we could identify two cases being misdiagnosed as IDC‐NST.

Experimentally, loss of Afadin in E‐cadherin‐expressing luminal‐type MCF7 cells leads to less confined cortical localization of F‐actin at the plasma membrane and subsequent colocalization with the E‐cadherin based AJ, indicative of incomplete maturation of the AJ. With the residual but aberrant localization of wildtype E‐cadherin complexes on the membrane, we conclude that the tumour suppressor function of Afadin might be due to an attenuated association to α‐catenin and the subsequent incomplete maturation of the E‐cadherin to F‐actin cytoskeleton link. In the case of Afadin loss, we found that the CC domain containing an α‐catenin and F‐actin binding region is necessary and sufficient to confer proper Actomyosin organisation at the AJ, even in the absence of the FAB domain. Because decreased cortical F‐actin localization and contraction are caused by a reduced buildup of forces along the F‐actin fibres connecting the cortical F‐actin to the AJ [52, 53], we conclude that this lack of mechanical tension is strongly exacerbated in the absence of Afadin, thus preventing apical constriction. Consequently, Rho kinase may be released from Shroom3 and drive Actomyosin contraction [54, 55], a process that will eventually drive the formation of metastatic tumours. One of the causes of the reduction in mechano‐transduction upon Afadin loss could be the forces of F‐actin exerted on Vinculin during initial AJ formation, which is essential for establishing the AJ and F‐actin connection [56]. It has become clear that α‐catenin is a rate‐limiting tension sensor between E‐cadherin and F‐actin [57] through its stretch‐dependent unfolding and binding to Vinculin [58]. Upon AJ maturation and following a balanced interplay of forces, localised expression levels of Vinculin are reduced in the junction, rendering a stabilised AJ including Afadin [59]. Previous work by Takai and co‐workers demonstrated that Afadin is required for proper AJ formation, but that the FAB domain is dispensable for this event [10]. Our work is in full agreement with these findings, showing that reconstitution of an Afadin truncate lacking the C‐terminal FAB domain fully rescues formation of the AJ and the restoration of an epithelial phenotype. In contrast, reconstitution with deletion truncates lacking the CC domain prevents cortical F‐actin colocalization with the E‐cadherin‐based AJ, which shows that direct association of the CC domain and Afadin upon AJ formation is key in establishing mechano‐responsive interactions to the F‐actin cytoskeleton.

It is nonetheless striking that the C‐terminal FAB domain is dispensable for proper AJ formation and function in breast cancer cells. Previous studies in *Drosophila* have demonstrated that, while the FAB domain is not essential for AJ formation, it supports the stability of AJ under tension [60]. In nonmalignant mammalian cells, loss of the FAB domain appears to delay the formation of adherens and tight junctions [38]. Interestingly, the CC domain of Afadin contains four predicted alpha helices, of which the first N‐terminal helix overlaps with the mapped binding site of Afadin to α‐catenin [61]. Recent analyses indicate that the predicted C‐terminal helices show a near complete overlap with the F‐actin binding site identified by Carminati *et al* [13], suggesting that the CC domain might not only confer α‐catenin binding, but also F‐actin interaction, or both [62]. In short, based on our work and the aforementioned studies, we propose that either: (i) the FAB domain binds F‐actin and enforces AJ stability but is not solely responsible for F‐actin linkage, (ii) all major functions related to AJ stability are deployed by Afadin through an α‐catenin dependent F‐actin linkage, or (iii) the predicted F‐actin binding sites in the CC intrinsically disordered region (IDR) are dominant in facilitating AJ formation and stability together with α‐catenin linkage (see Figure 6). A final and alternative option comes from the recent finding that a direct interaction between Afadin and α‐catenin may not be required for maturation of the normal epithelial junction [63]. In this scenario, apical constriction in epithelial cells relies on two distinct constriction belts named the *zonula adherens matura*, for which Afadin is essential and where the main F‐actin contraction converges on a Nectin–Afadin interphase. Future work will have to delineate the exact mechanisms that underpin the role of Afadin in AJ formation and function through fine‐mapping of the predicted F‐actin sites with the IDR of Afadin.

In conclusion, we demonstrate that Afadin loss leads to an F‐actin dependent noncohesive cellular phenotype, founded in detrimental cortical Actomyosin dynamics that result in anchorage independence and metastatic dissemination. As such, we reaffirm the proposition that carcinomas driven by loss of the AJ, or the loss of the F‐actin cytoskeleton connection with the AJ, should be considered ‘Actin’‐driven diseases [47]. Delineating the disruption of proper AJ formation will be key in the correct diagnosis and future treatment of malignancies driven by functional loss of E‐cadherin such as ILC and diffuse gastric cancer.

## Author contributions statement

MAKR and PWBD designed the study. MAKR, TK, and PWBD wrote the article. MAKR, LNFLE, SvK, CHJV, WEH and TK performed experiments. LNFLE and MAKR performed preclinical mouse experiments. PWBD and IJN designed the targeted sequencing capture strategy and performed subsequent analyses. RB and CC provided patient data, tissues and DNA samples. NI provided conceptual input to define Afadin form, function, and cloning of the Afadin truncate constructs. MC and PJvD performed human and mouse histological tumour diagnostics. All authors read the article and were given the opportunity to provide input.

## Supporting information


**Figure S1.** Somatic *AFDN* mutations in breast cancer
**Figure S2.** Expression of E‐cadherin, β‐catenin, α‐catenin and ZO1 in Afadin knockout MCF7 cells
**Figure S3.** Afadin loss in a primary breast cancer tumour model

**Table S1.** The Adhesome gene list

**Table S2.** TCGA & METABRIC diagnosis, *AFDN* and *CDH1* status

**Table S3.** Guide RNAs and primers used

## Data Availability

The data that support the findings of this study are available on request from the corresponding author. The data are not publicly available due to privacy or ethical restrictions.
