# Supplementary material for: Afadin loss induces breast cancer metastasis through destabilisation of E‐cadherin to F‐actin linkage
Source: J Pathol. 2025 Mar 3;266(1):26–39. doi: 10.1002/path.6394 (PMC11985701; doi:10.1002/path.6394)
Supplement: Supplementary file 1 — Figure S1. Somatic AFDN mutations in breast cancer Figure S2. Expression of E‐cadherin, β‐catenin, α‐catenin and ZO1 in Afadin knockout MCF7 cells Figure S3. Afadin loss in a primary breast cancer tumour model [file PATH-266-26-s002.docx]

**Afadin loss induces breast cancer metastasis through destabilization of E-cadherin to F-actin linkage**

MAK Rätze *et al. J Pathol* <https://doi.org/10.1002/path.6394>

**Supplementary Figures S1–S3**

**Supplementary Tables S1–S3 are provided as supplementary Excel files**

**Supplementary Figures S1–S3**

**Figure S1.** Somatic *AFDN* mutations in breast cancer. (A) Lollypop plot depicting the distribution of the specific mutation types (coloured bullets) and incidence (lollypop length) across the *AFDN* coding region. Data were retrieved from cBioportal (METABRIC and TCGA datasets). Different aa=amino acids; IDC-NST=invasive ductal carcinoma of no specific type; ILC=invasive lobular carcinoma; unknown=unknown breast cancer type. (B) *AFDN* and *CDH1* somatic mutations are mutually exclusive in ILC. The table is showing the number of mutations found in the ductal, lobular or mixed histological breast cancer types in TCGA and METABRIC. Significant mutual exclusivity for inactivating mutations in *AFDN* and *CDH1* is found in the ILC and Mixed breast cancers.

**Figure S2. Expression of E-cadherin, β-catenin, α-catenin, and ZO1 in Afadin knockout MCF7 cells.** (A–C) Immunofluorescence images showing single and merged images (right panels) of E-cadherin and α-catenin (A), E-cadherin and β-catenin (B), and afadin and ZO-1 (C) in MCF7 wildtype (Wildtype) and MCF7::∆*AFDN* cells (∆*AFDN*). Note the loss of ZO-1 expression at the tricellular junctions. Scale bar = 5 µm.

**Figure S3. Afadin loss in a primary breast cancer tumour model.** (A) Afadin was knockedout in the primary breast cancer organoid 209T (HUB-01-C2-156) and verified by western blotting using an afadin targeting antibody. The * denotes a-specific bands that were used as loading controls. (B,C) *AFDN* knockout induces loss of organoid integrity in the 209T model in 3D. Control (wildtype) and *AFDN* knockout cells (209T::∆*AFDN*#1, or 209T::∆*AFDN*#2) were cultured in BME and photographed (note the acquisition of the irregular organoid shaped in the knockout PDOs) in panel (B). PDOs were also subjected to immunofluorescence for afadin (green), E-cadherin (red), and F-actin (white). A merged image is shown in the right panels (C). Scale bars = 25 µm.
